# Revisiting the role of cancer-associated fibroblasts in tumor microenvironment

**DOI:** 10.3389/fimmu.2025.1582532

**Published:** 2025-04-17

**Authors:** Xiaolei Lan, Wenyang Li, Kai Zhao, Jianpeng Wang, Shifang Li, Hai Zhao

**Affiliations:** Department of Neurosurgery, The Affiliated Hospital of Qingdao University, Qingdao, Shandong, China

**Keywords:** cancer-associated fibroblasts, tumor microenvironment, therapeutic resistance, biomarkers, immune modulation, targeted therapies

## Abstract

Cancer-associated fibroblasts (CAFs) are integral components of the tumor microenvironment playing key roles in tumor progression, metastasis, and therapeutic resistance. However, challenges persist in understanding their heterogeneity, origin, and functional diversity. One major obstacle is the lack of standardized naming conventions for CAF subpopulations, with current systems failing to capture their full complexity. Additionally, the identification of CAFs is hindered by the absence of specific biomarkers, limiting the precision of diagnostic and therapeutic strategies. *In vitro* culture conditions often fail to maintain the *in vivo* characteristics of CAFs, which complicates their study and the translation of findings to clinical practice. Although current detection methods, such as antibodies, mRNA probes, and single-cell transcriptomics, offer insights into CAF biology, they lack standardization and fail to provide reliable quantitative measures. Furthermore, the dynamic interactions between CAFs, tumor cells, and immune cells within the TME remain insufficiently understood, and the role of CAFs in immune evasion and therapy resistance is an area of ongoing research. Understanding how CAFs influence drug resistance and the immune response is essential for developing more effective cancer therapies. This review aims to provide an in-depth analysis of the challenges in CAF research, propose future research directions, and emphasize the need for improved CAF-targeted therapeutic strategies. By addressing these gaps, it seeks to highlight the potential of CAFs as targets for overcoming therapeutic resistance and enhancing the efficacy of cancer treatments.

## Introduction

Cancer-associated fibroblasts (CAFs) are a critical component of the tumor microenvironment (TME) and play pivotal roles in cancer progression, metastasis, and therapeutic resistance (1–3). As the most abundant stromal cells within tumors, CAFs are involved in various processes that facilitate tumor growth, including remodeling of the extracellular matrix (ECM), promoting angiogenesis, and influencing immune responses. Additionally, CAFs contribute to the metabolic reprogramming of the tumor, establishing a pro-tumorigenic microenvironment (4). Recent research has highlighted the complex and often dualistic nature of CAFs, as they can exhibit both tumor-promoting and tumor-suppressing activities depending on their activation state, subpopulations, and interactions within the TME (2).

Despite their central role in cancer biology, significant challenges remain in understanding CAFs due to their heterogeneity, dynamic plasticity, and the lack of specific markers for accurate identification (5). The classification of CAF subpopulations is further complicated by the absence of standardized naming conventions, making it difficult to compare findings across studies (2). Moreover, CAFs exhibit a high degree of functional diversity that varies between different tumor types and stages, complicating efforts to develop universal therapeutic strategies targeting CAFs (6). Their ability to modulate immune responses, promote resistance to conventional therapies, and contribute to immune evasion has made them a significant target in cancer treatment (7). For instance, CAFs can inhibit the infiltration of immune cells into the tumor, enhance the immunosuppressive microenvironment, and reduce the efficacy of both chemotherapy and immunotherapy.

The potential for CAF-targeted therapies is considerable, but several obstacles remain, including the difficulty in isolating CAFs and maintaining their characteristics *in vivo* and *in vitro* (4). Furthermore, understanding the molecular mechanisms underlying CAF interactions with tumor cells, immune cells, and the ECM is essential for the development of effective CAF-targeted treatments. This review aims to provide a comprehensive analysis of the current challenges in CAF research, exploring their roles in tumor progression, therapeutic resistance, and immune modulation, while also discussing promising future directions for targeted therapies that aim to overcome the barriers CAFs present in cancer treatment.

## Fibroblasts and CAFs

**Figure 1:**
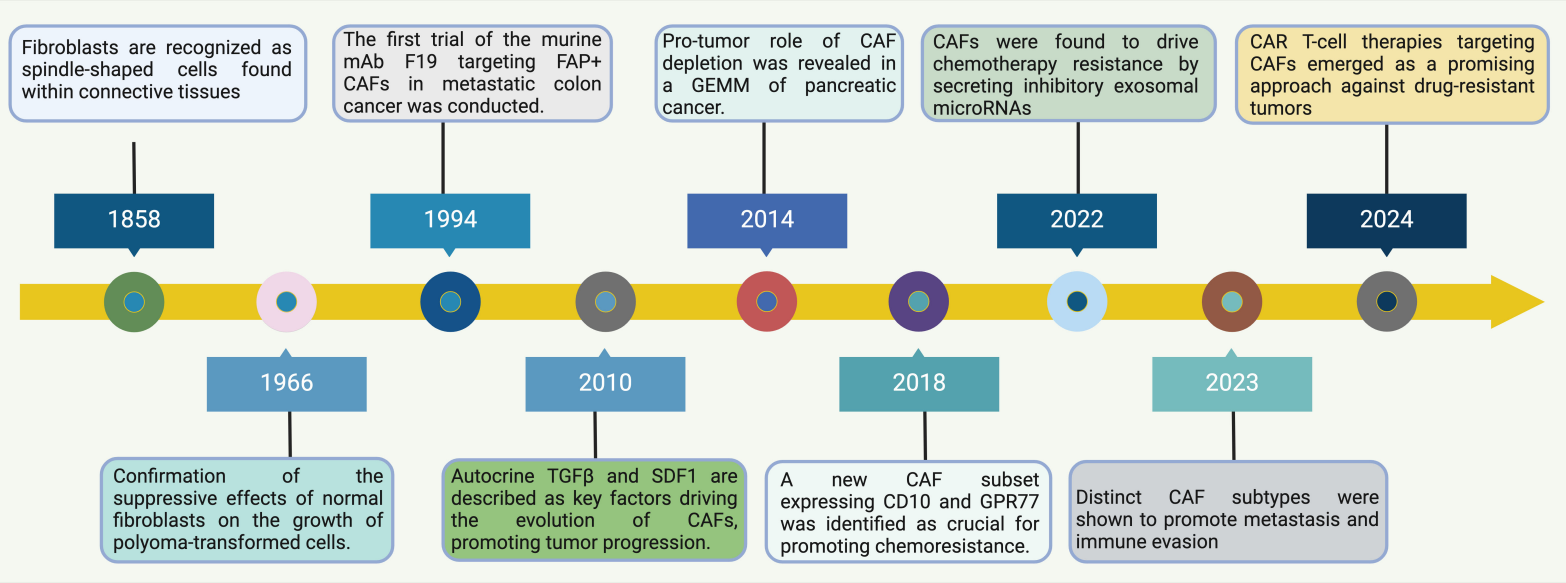
Timeline of Discoveries in Normal Fibroblasts and CAFs. This timeline illustrates significant milestones and the evolving focus of research on fibroblasts, especially in relation to their role in cancer. Starting from the historical identification of fibroblasts as spindle-shaped cells in connective tissues in 1858, the understanding of CAFs has evolved significantly (15). By 1966, research confirmed the suppressive effects of normal fibroblasts on the growth of polyoma-transformed cells, highlighting a functional aspect of fibroblasts in modulating cellular transformation (16). Progressing through the timeline, by 1994, the first trial targeting FAP+ CAFs using murine mAb F19 in metastatic colon cancer was conducted, illustrating a move towards targeted CAF therapies (17). In 2010, autocrine factors like TGFβ and SDF1 were identified as key drivers in the evolution of CAFs, promoting tumor progression, showcasing the increasing understanding of the molecular mechanisms by which CAFs influence cancer dynamics (18). Further advances in 2014 highlighted the role of CAFs in the depletion processes in a genetically engineered mouse model of pancreatic cancer, signifying the impact of CAFs beyond the tumor cells themselves (19). By 2018, the identification of a new CAF subset expressing CD10 and GPR77 was crucial for understanding chemoresistance, pointing towards the heterogeneity within CAF populations and their varied roles in cancer progression (20). Recent discoveries in 2022 and 2023 have established that CAFs drive chemotherapy resistance by secreting inhibitory exosomal microRNAs and promote metastasis and immune evasion through distinct CAF subtypes, respectively (21). These findings underscore the multifaceted roles of CAFs in facilitating tumor aggression and resisting therapeutic interventions. Finally, CAR T-cell therapies targeting CAFs have emerged as a promising approach against drug-resistant tumors, marking a pivotal shift towards leveraging immune system modifications to combat the complex interactions within the TME driven by CAFs (22). This shift not only represents a novel therapeutic strategy but also highlights the ongoing evolution of understanding in the field of tumor immunology and the critical role CAFs play in shaping cancer outcomes. Abbreviations: CAF, Cancer-Associated Fibroblast; FAP, Fibroblast Activation Protein; GEMM, Genetically Engineered Mouse Model; CAR, Chimeric Antigen Receptor; TGF, Transforming Growth Factor; SDF1, Stromal Cell-Derived Factor 1; GPR77, G Protein-Coupled Receptor 77.

Despite ongoing discussions about the precise definition of fibroblasts, they were originally identified about 150 years ago as spindle-shaped cells that produce collagen in connective tissues (8) **(** **Figure 1** **)**. Current evidence suggests that fibroblasts in healthy tissues are dormant mesenchymal cells located within the extracellular matrix’s interstitial fibers. These cells can become activated in response to specific conditions such as wound healing, tissue inflammation, and organ fibrosis. In the context of cancer, analogous processes include cancer development (referred to as the ‘cancer wound’), inflammation that promotes tumor growth, and tumor-associated fibrosis (9–11). Activated fibroblasts in cancer settings are variously known as CAFs, tumor-associated fibroblasts, peritumoral fibroblasts, myofibroblasts, or reactive stromal fibroblasts (12, 13). These CAFs adapt alongside cancer cells, adopting a pro-tumor phenotype that allows them to thrive and expand within the complex tumor microenvironment, thereby facilitating tumor progression (14).

Fibroblasts are spindle-shaped, non-epithelial, non-immune cells embedded within the extracellular matrix (ECM) that are easily propagated in adherent cell culture (23, 24). They play a key role in the stroma of gastrointestinal organs, where they are well-organized, much like in other tissues. Throughout the gastrointestinal tract, a reticular network of stromal cells is closely associated with the epithelial basement membrane (25). The subepithelial plexus, consisting of reticular stromal cells, completely surrounds the glandular axis from the stomach to the rectum (26). This compartment is dynamic, with a radial axis of proliferation and differentiation similar to that of the epithelium, originating from gremlin 1-expressing intestinal reticular stem cells (27). These stem cells give rise to intestinal reticular cells, likely overlapping with FOXL1+ subepithelial telocytes and GLI1+ mesenchymal cells, which together form an essential mesenchymal niche supporting intestinal stem cells (26–28). Beneath this compartment is a loosely organized network of fibroblasts within the lamina propria that interact with one another as well as with deeper stromal elements, including smooth muscle, blood vessels, nerves, and inflammatory cells (25, 29, 30). Functionally, fibroblasts are crucial in regulating ECM synthesis and facilitating paracrine and juxtacrine signaling to adjacent epithelial cells, thereby influencing their growth and differentiation (23, 31). They are also poised to respond to tissue damage, whether due to injury or tumorigenesis.

Cancer-associated fibroblasts (CAFs) are broadly recognized as the fibroblasts located within and surrounding tumors (14). This group comprises native, normal fibroblasts, as well as activated, proliferating (Ki67+) or recruited fibroblasts in response to cancer-derived stimuli. These newly formed CAFs may arise through several mechanisms, which will be discussed later. Despite the advancements in immune cell immunophenotyping and subtyping, a definitive marker for CAFs remains elusive (14, 32, 33). This gap has led to the identification of overlapping, incomplete, or distinct CAF populations in different studies, with markers that label both CAFs and other cell types. These challenges have complicated the interpretation of many studies, which will be addressed further.

## The origin of CAFs

**Figure 2:**
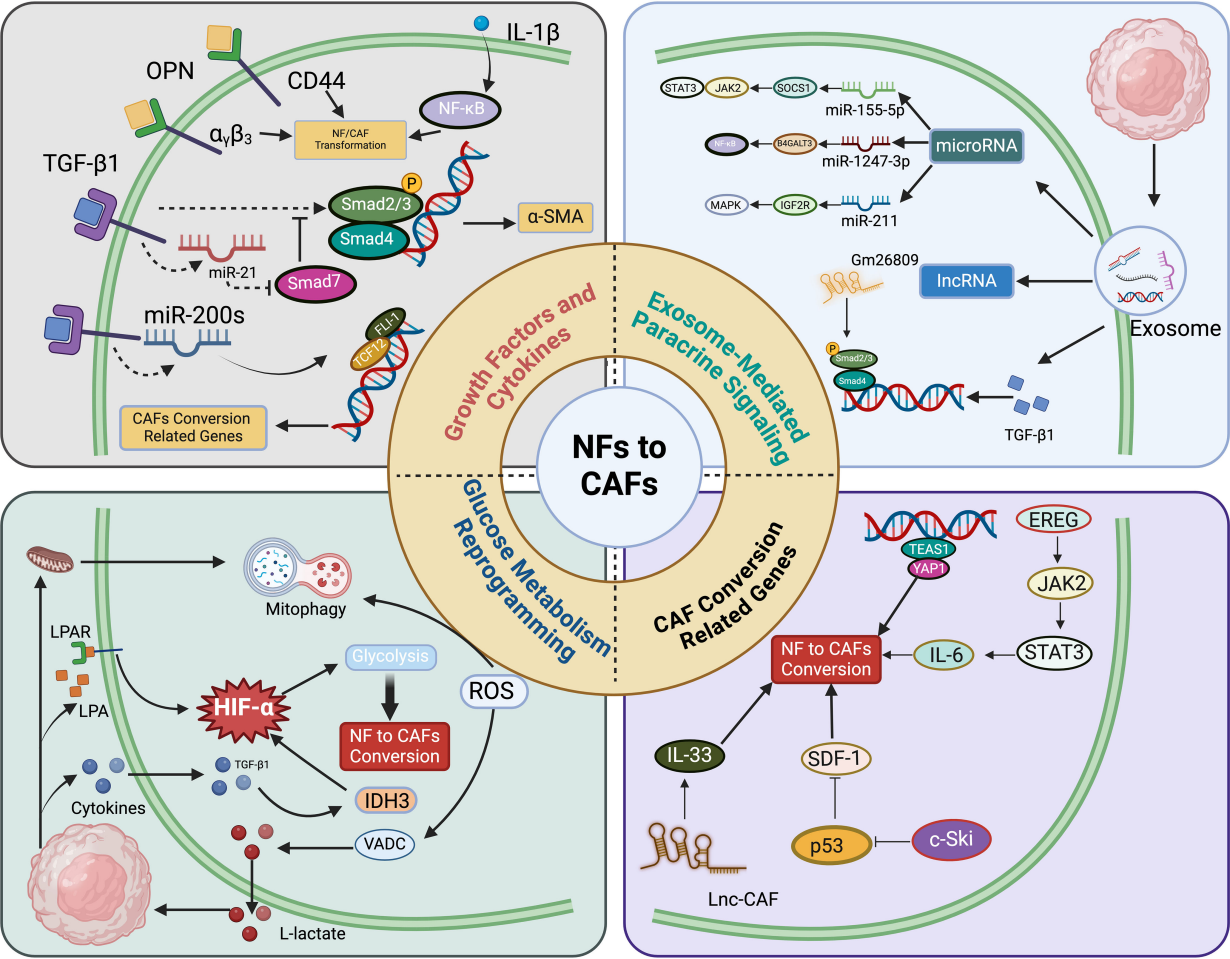
Conversion from normal fibroblasts (NFs) to cancer-associated fibroblasts (CAFs) involves multiple molecular mechanisms. a) Growth factors and cytokines like transforming growth factor-beta 1 (TGF-β1), osteopontin (OPN), and IL-1β interact with their respective receptors in NFs, subsequently activating downstream effectors such as miRNAs and CD44 (61–63). These molecules modulate the expression of genes associated with the CAF phenotype through the TGF-β/Smads and NF-κB signaling pathways (64, 65). b) Exosomes derived from cancer cells, carrying entities such as miRNAs and lncRNAs, induce the transformation of NFs into CAFs (65). This conversion is facilitated by signaling pathways including TGF-β/Smads, JAK/STAT, NF-κB, and MAPK cascades. c) The shift from NF to CAF is also driven by alterations in glucose metabolism, with the HIF-1α signaling pathway playing a crucial role in this metabolic reprogramming (66). d) Variations in cellular homeostasis prompt a self-driven transition to CAFs by regulating the activation of cytoskeletal proteins and the secreted phenotype, primarily through the JAK/STAT and p53 signaling pathways (67).

Due to the absence of specific biomarkers, identifying the origin of CAFs remains challenging. Current evidence primarily supports that fibroblasts originate from primitive mesenchymal cells, while CAFs arise from activated fibroblasts within local tissues (34, 35). Studies have shown that normal fibroblasts can proliferate, become activated, and express CAF markers by internalizing exosomes released from bladder cancer cells. Further research indicates that bladder cancer cells transform normal fibroblasts into CAFs through exosome-mediated transmission of transforming growth factor-beta (TGF-β) and SMAD signaling pathways (35, 36). **Figure 2** illustrates the intricate and multifactorial processes involved in the transformation from normal fibroblasts (NFs) to CAFs. The recent research highlights the roles of key molecules and pathways, including transforming growth factor-beta 1 (TGF-β1), osteopontin, and interleukin-1β (IL-1β) (37–39). These elements initiate the transformation by engaging their respective receptors on NFs, subsequently activating downstream signaling cascades such as TGF-β/Smads and NF-κB, pivotal for modulating gene expression linked to the CAF phenotype (40). Exosomes from cancer cells, carrying miRNAs and lncRNAs, significantly contribute to the transformation of NFs into CAFs, mediated by pathways including TGF-β/Smads, JAK/STAT, and MAPK (41–43). These exosomes facilitate a feedback loop that enhances the conversion process. Additionally, the diagram delineates how alterations in glucose metabolism, driven by the hypoxia-inducible factor-1α (HIF-1α) pathway, are crucial for metabolic reprogramming essential for the survival and function of CAFs (44). HIF-1α are stabilized through both hypoxia-dependent mechanisms, where low oxygen levels inhibit their degradation, and hypoxia-independent pathways, such as activation by growth factors that affect the proteasome pathway (45). Key target genes involved in the transition from NFs to CAFs include VEGF for angiogenesis, GLUT1 for glucose metabolism, LOX for extracellular matrix remodeling, and CA9 for pH regulation (42, 46, 47). These genes contribute to the CAF phenotype, enhancing their ability to support tumor progression. Moreover, the transition to a CAF phenotype is driven by changes in cellular homeostasis, regulated by the activation of cytoskeletal proteins and the secreted phenotype, primarily through JAK/STAT and p53 signaling pathways. This comprehensive portrayal underscores the dynamic network of interactions that define CAF biology **(** **Figure 2** **).**

Additionally, a novel microfluidic model has been developed to regulate the three-dimensional tumor microenvironment (TME) *in vitro*, revealing that exosomes derived from melanoma can drive the differentiation of endothelial cells into CAFs through endothelial-mesenchymal transition (EndMT). Moreover, exosomes derived from mesenchymal stem cells (MSCs) have been shown to inhibit EndMT and induce CAFs to undergo reverse differentiation back into endothelial cells (48). This suggests that exosomes with the ability to reverse CAF differentiation could serve as effective carriers for anti-tumor drugs. Furthermore, epithelial cells in the TME can differentiate into CAFs via epithelial-mesenchymal transition (EMT) (49). Subsequently, CAFs secrete cytokines that promote EMT in tumor cells, ultimately facilitating tumor invasion and metastasis (50). Other studies have demonstrated that TGFβ1 can induce the differentiation of bone marrow-derived MSCs into CAFs by activating the JAK/STAT3 signaling pathway, promoting the migration and invasion of colorectal cancer cells (51). Various studies also support that PDGFα-CAF cells originate from MSCs (52). Additionally, several other sources of CAFs have been identified, including hematopoietic stem cells (HSCs), cancer stem cells (CSCs), adipocytes, pericytes, and stellate cells (47, 53–60). However, there is limited evidence supporting these origins, and their relevance to different tumor types remains uncertain.

**Figure 3:**
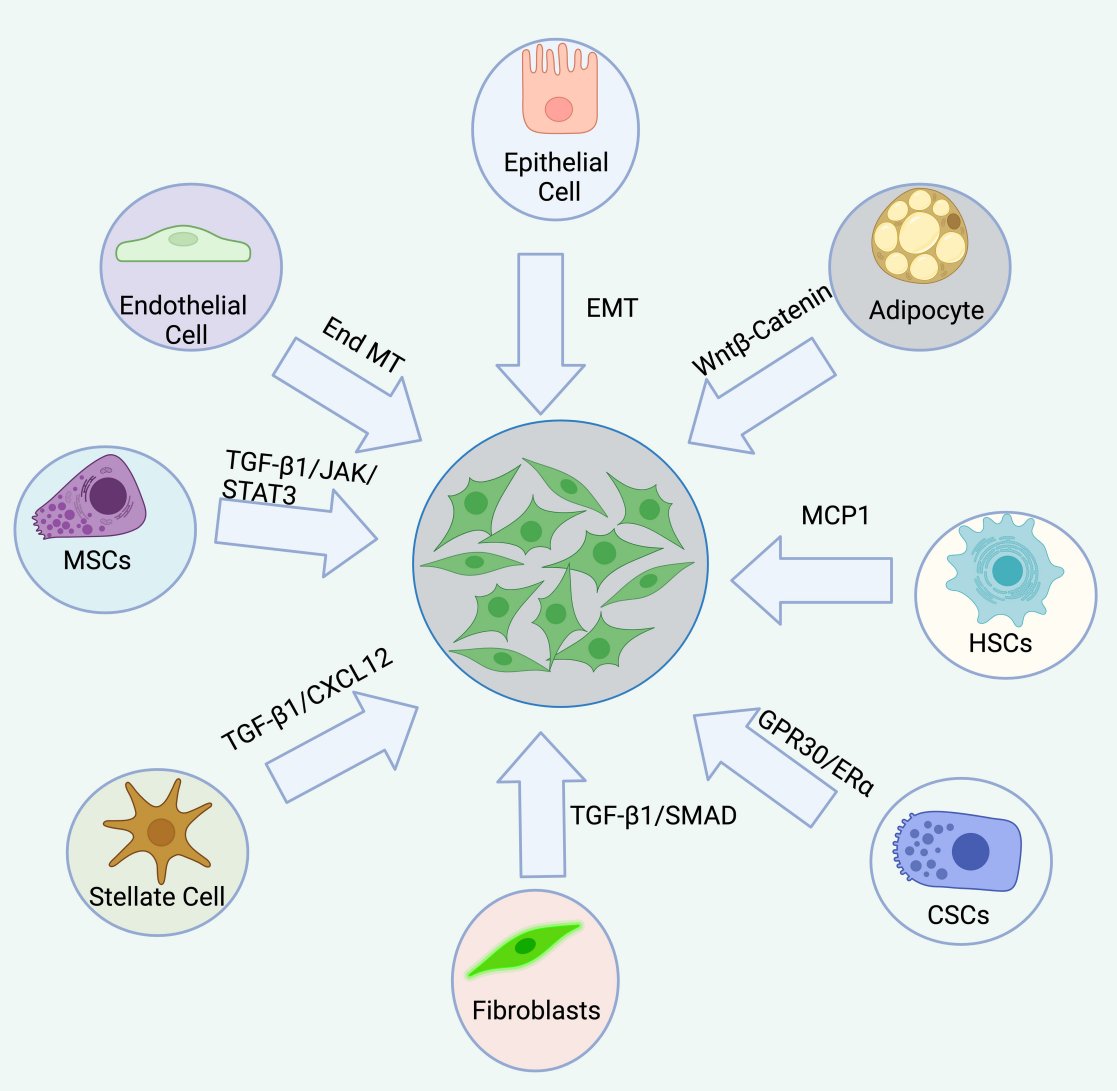
The origin of CAFs are complex, diverse, and heterogeneous. CAFs originate from a variety of sources, including resident fibroblasts, epithelial cells, endothelial cells, bone marrow-derived mesenchymal stem cells (MSCs), hematopoietic stem cells (HSCs), cancer stem cells (CSCs), adipocytes, pericytes, and stellate cells. Each origin is associated with distinct signaling pathways.

In summary, CAFs originate from fibroblasts, epithelial cells, endothelial cells, bone marrow-derived MSCs, HSCs, CSCs, adipocytes, pericytes, and stellate cells (47, 53–60). This diversity in origin contributes to the heterogeneity of CAFs, with different origins being regulated by distinct signals or factors, resulting in varied differentiation pathways. Further research into CAF origins may provide insights into the discovery of biomarkers, therapeutic targets, signaling pathways, and activation mechanisms, all of which hold significant clinical potential. However, regardless of their cellular origin, the state, phenotype, and function of CAFs dynamically evolve throughout tumor progression, varying across different pathological stages (**Figure 3**).

CAFs originate from a variety of sources, including resident fibroblasts, epithelial cells, endothelial cells, bone marrow-derived mesenchymal stem cells (MSCs), hematopoietic stem cells (HSCs), cancer stem cells (CSCs), adipocytes, pericytes, and stellate cells. Each origin is associated with distinct signaling pathways.Common CAF markers and gene signatures

In general, normal fibroblasts exhibit significant heterogeneity, which is reflected in differences in their morphology, behavior, and gene expression (as reviewed in (68)). In addition to the intrinsic heterogeneity within fibroblasts, other stromal cells share similarities with fibroblasts, particularly in the expression of so-called ‘specific’ markers. As a result, the complexity of the stromal compartment, along with the existence of multiple fibroblast subpopulations, makes it challenging to accurately isolate and define CAFs based on specific marker expression. Despite these challenges, numerous CAF markers are well-documented in the literature. Among the downregulated markers are caveolin-1 (69) and laminin (70), while frequently reported upregulated CAF markers include alpha-smooth muscle actin (aSMA) (71), vimentin (72), and fibroblast-specific protein 1 (FSP1) (32). However, Sugimoto et al., using a cancer mouse model, compared six markers from various mesenchymal cell groups residing in the stroma. They found that aSMA, platelet-derived growth factor receptor beta (PDGFRβ), and chondroitin sulfate proteoglycan 4 were expressed by fibroblasts, myofibroblasts, and vascular-associated cells, while vimentin and type I collagen also lacked specificity for fibroblasts (32). These findings raise questions about the specificity of these commonly accepted CAF markers. Moreover, aSMA was found to be downregulated, rather than upregulated, in the stromal compartment of prostate cancer (73). This highlights the heterogeneity within the stromal compartment and suggests that no definitive marker exists to clearly distinguish between mesenchymal subpopulations. Despite the fact that aSMA is often elevated in a cancer-type-specific manner and is considered a general marker for mesenchymal cells, it is incorrectly regarded as a universal marker for all CAFs (12, 74).

Several studies have identified a ‘CAF gene expression profile’ (75–77) and even ‘CAF signatures’ that can predict tumor outcomes (78, 79). A closer analysis of these studies reveals that, at the individual gene level, the differences between these profiles outweigh the similarities. However, when viewed from a broader perspective, examining gene families rather than individual genes, different cancer types and stages reveal similar groups of genes involved in processes such as cell adhesion, immune response, and extracellular matrix (ECM) modulation (75, 80, 81). This pattern aligns with expectations, as various cell types exposed to similar conditions tend to converge in performing the same functional tasks, albeit through different sets of genes.

## Function of CAFs

The functions of CAFs has employed a range of methodologies from basic cell culture experiments and animal models to correlational studies within extensive patient groups. These methodologies have disclosed a broad spectrum of CAF functions. The relatively straightforward process of culturing CAFs and corresponding normal fibroblasts from patient samples has significantly advanced the understanding of CAF mechanisms. Notably, CAFs are extremely efficient at depositing and remodeling the extracellular matrix (ECM) in the tumor microenvironment. This capability is regulated by RHO and RAB GTPase-mediated mechanisms controlling integrin-linked adhesions and the actomyosin cytoskeleton, which is associated with the suppression of the CD36 transmembrane receptor (82–85). Furthermore, CAFs synthesize enzymes that form matrix crosslinks and engage in force-driven ECM remodeling, contributing to the increased rigidity of tumor tissues. Although these chemical crosslinks are stable, the secretion of matrix proteases by CAFs facilitates dynamic remodeling of the tumor matrix, creating pathways that enable cancer cell migration. Eph–ephrin signaling mediated by direct cell contact also affects cancer cell motility (86). Beyond fostering local invasion, CAFs enhance metastasis in experimental settings, a process linked to their ECM remodeling activities (87–90). At secondary metastatic sites, newly activated fibroblasts support the development of extensive metastases through various mechanisms, including the production of matrix molecules like tenascin and periostin, which fortify cancer cell signaling via WNT pathways (91–93). Recent findings suggest that alterations in ECM structure can also impact the migration of immune cells, with significant implications for tumor immune surveillance (90, 94, 95).

Alterations in matrix production and tumor mechanics driven largely by CAF activities have multifaceted implications for cancer. Enhanced tissue stiffness activates signaling pathways in cancer cells that promote survival and proliferation (96). Additionally, increased mechanical stress can compress blood vessels, inducing hypoxia, which fosters more aggressive cancer phenotypes and hampers drug delivery (97–99). Changes in tissue mechanics might also play a role in the onset of cancer and pre-malignant conditions, as evidenced by the relationship between mammographic density and breast cancer risk (85). Strategies aimed at disrupting the interactions between CAFs and the mechanical properties of tumors for therapeutic benefit are currently under investigation.

CAFs are also significant producers of growth factors, cytokines, and exosomes that encourage tumor growth and influence responses to therapy (42, 100, 101). They secrete TGF-β, leukemia inhibitory factor (LIF), growth arrest-specific protein 6 (GAS6), fibroblast growth factor 5 (FGF5), growth differentiation factor 15 (GDF15), and hepatocyte growth factor (HGF), which drive the invasive and proliferative behaviors in cancer cells (102–107). HGF, in particular, is noted for promoting resistance to BRAF-targeted therapies by activating an alternative BRAF-independent pathway for ERK–MAPK signaling (108, 109).

The secretome of CAFs additionally affects other components of the tumor microenvironment (110–114). VEGF from stromal cells can initiate angiogenesis, while a variety of cytokines and chemokines from CAFs act on different leukocytes, including CD8+ T cells, regulatory T cells, and macrophages, yielding both suppressive and promotional immune responses (114). However, the overarching impact of CAFs is immunosuppressive, mediated by factors like IL-6, CXCL9, and TGFβ, which notably reduce T cell activity (115). Recent observations of antigen cross-presentation by CAFs have highlighted their role in modulating CD4+ and CD8+ T cell responses (116, 117). Clinical studies further corroborate a negative correlation between CAF presence and CD8+ T cell levels (118). Moreover, IL-6 may also foster systemic immuno-suppression through metabolic effects (119). Disrupting CXCL12 from CAFs enhances T cell-mediated tumor control, and targeting focal adhesion kinase (FAK) in cancer cells simultaneously reduces stromal fibroblast activation and the formation of an immunosuppressive milieu (120). Nonetheless, the role of tumor necrosis factor (TNF) produced by CAFs is complex; while TNF can activate fibroblasts, its tumor-promoting, immunosuppressive effects are tied to the suppression of TNF signaling (121, 122).

Lastly, the metabolic exchange between cancer cells and CAFs provides another layer of interaction, where autophagy in stromal fibroblasts produces alanine used by pancreatic ductal adenocarcinoma cells to energize the tricarboxylic acid cycle (123–125). Additionally, metabolic irregularities in CAFs may link to altered immune regulation, potentially through IL-6 production or the depletion of immunomodulatory amino acids (126).

## CAFs in cancer

CAFs play a multifaceted role in cancer progression and metastasis by fostering a conducive tumor microenvironment through several mechanisms (103, 127, 128). Firstly, CAFs contribute to cancer cell stemness and metastatic capabilities by engaging in paracrine signaling with cancer stem cells, enhancing their self-renewal and propagation abilities which are crucial for tumor aggressiveness and metastasis (42, 129). Secondly, they promote tumor angiogenesis by secreting pro-angiogenic factors like VEGFA and PDGF and altering the extracellular matrix to enhance vascular formation, facilitating tumor growth and the dissemination of cancer cells (130–132). Thirdly, CAFs are instrumental in mediating immunosuppression within the tumor microenvironment by secreting factors such as TGF-β and IL-6, which modulate immune cell function and contribute to immune evasion by the tumor (133–135). Lastly, they are involved in metabolic reprogramming known as the “Reverse Warburg Effect,” where they supply cancer cells with metabolic intermediates necessary for energy production, thus supporting the energetic demands of rapidly proliferating tumor cells (136, 137). Through these interactions, CAFs are key players in enabling cancer progression and the establishment of metastatic sites, making them significant targets for therapeutic intervention.

### CAFs contribute to cancer stemness

CAFs are integral to the tumor microenvironment (TME) and play a pivotal role in the maintenance and enhancement of cancer cell stemness, characterized by self-renewal and the ability to propagate, which are key traits of cancer stem cells (CSCs) (138–140). These cells are known to drive tumor aggression, contribute to resistance against therapies, and facilitate metastasis. CSCs are identified through various markers such as CD44, CD24, CD133, LGR5, SOX2, AQP5, ESA, PAF1, and CXCR4, though these markers lack high specificity (141). CAFs interact with CSCs predominantly through paracrine signaling, supporting a conducive niche for tumor growth (142). Research has highlighted that certain CAF subsets secrete molecules that directly enhance CSC properties. For example, a specific subset of CAFs expressing CD10 and GPR77, activated by NF-kB, has been shown to enrich CSCs in breast and lung cancers by releasing IL-6 and IL-8 (20). Similarly, in bladder cancer, CAFs stimulated by interferon from cancer cells can enhance stemness through the WNT5a/β-catenin signaling pathway (143). In the context of hepatocellular carcinoma, CAFs boost cancer cell stemness via the ERK1/2-FRA1-HEY1 pathway by secreting hepatocyte growth factor (144). Additionally, CAFs are known to produce exosomes that perpetuate stemness across various cancer types (145, 146). They also indirectly facilitate the recruitment and stemness of myeloid-derived suppressor cells through FAP-dependent mechanisms (146). Given the significant role of these paracrine interactions, targeting such pathways might offer new therapeutic avenues, particularly through manipulation of WNT signaling which is crucial in mediating interactions between CAFs and CSCs, affecting both the active and dormant CSCs (147).

### CAFs promote angiogenesis

Angiogenesis, the formation of new blood vessels, is essential for tumors to secure a greater supply of oxygen and nutrients. This process is predominantly driven by hypoxia within the tumor environment. Under these low-oxygen conditions, cancer cells release vascular endothelial growth factor A (VEGFA), which targets VEGF receptor 2 (VEGFR2) on adjacent endothelial cells (ECs) or on circulating endothelial progenitor cells derived from the bone marrow, thus initiating angiogenesis (148). This angiogenic cascade includes degradation of the basal lamina and extracellular matrix, EC proliferation, the development of vascular sprouts, and eventual vessel stabilization. Additionally, molecules like delta-like ligand 4 (DLL4) and angiopoietin 2 (ANGPT2) are vital for angiogenesis (149).

CAFs secrete a range of pro-angiogenic growth factors such as VEGFA, CXC-chemokine ligand 12 (CXCL12), fibroblast growth factor 2 (FGF2), and platelet-derived growth factor (PDGF) (150). CXCL12, also recognized as stromal cell-derived factor 1 (SDF-1), promotes tumor proliferation and angiogenesis via the CXCL12/CXCR4 pathway (151). This interaction triggers diverse signaling pathways, including the G-protein coupled/PI3K/AKT/NF-κB axis and the Ras-MEK1/2-Erk1/2 axis, leading to angiogenic responses (152). FGF2, part of the heparin-binding growth factor family, engages FGF receptors to stimulate multiple angiogenic activities and interacts with the VEGF pathway (153). Moreover, the PDGF/PDGFR signaling pathway is crucial in the development of connective tissue and wound healing, with studies indicating that PDGF-C upregulation in CAFs can promote angiogenesis even in the absence of VEGF activity (154, 155).

Beyond direct activation through paracrine signaling, CAFs also indirectly enhance angiogenesis via biomechanical properties of the tumor microenvironment, such as matrix stiffness (156). CAFs produce enzymes like lysyl oxidase (LOX) and lysyl hydroxylase 2 (LH2), which increase collagen and elastin cross-linking, thus raising matrix stiffness (157, 158). Research has shown that higher matrix stiffness correlates with enhanced VEGF binding by endothelial cells, which is influenced by β1 integrins (159). This relationship extends to complex pathways involving Ca2+ influx and HIF-1α ubiquitination in hepatocellular carcinoma angiogenesis, highlighting the multifaceted role of the ECM in angiogenic regulation (160). However, conflicting findings in studies like those by Bao et al., which suggest that increased stiffness may suppress VEGF secretion in certain cancer types, underscore the complexity of these interactions and the need for more nuanced research (161).

### CAFs dedicate in metabolic changes in cancer

Despite residing in a nutrient-limited tumor microenvironment (TME), cancer cells demonstrate a remarkable capability for continuous proliferation, aided by metabolic adaptations within the TME. Notably, Warburg et al., about a century ago, documented that cancer cells preferentially convert glucose to lactate to generate ATP—even in the presence of sufficient oxygen—a process now known as the “*Warburg Effect*” *(* 162). Warburg attributed this metabolic peculiarity to mitochondrial dysfunction in cancer cells. However, subsequent studies in cancer metabolism have revealed that not all tumor cells are wholly reliant on this pathway; some retain mitochondrial functionality and can engage in oxidative phosphorylation (OXPHOS), illustrating the Warburg Effect’s variability across different tumor environments (163).

This reverse *Warburg Effect* is primarily propelled by oxidative stress induced by cancer cells. The release of reactive oxygen species (ROS) by cancer cells heightens oxidative stress in stromal components, causing autophagosomes to merge with lysosomes, which leads to mitochondrial degradation in CAFs. This process also results in the breakdown of caveolin-1 (Cav-1) via the HIF-1α/NF-κB pathway (136). The subsequent reduction of Cav-1 in CAFs further increases ROS in cancer cells, fostering a feedback loop that amplifies oxidative stress and disrupts the NF-kB pathway (164). Additionally, TGF-β, a key regulator in cancer metabolism, influences ROS levels by modulating the expression of α-SMA and NOX4 in fibroblasts, thereby promoting oxidative stress (165–167).

Through the reverse *Warburg Effect*, CAFs under oxidative stress due to cancer-derived ROS undergo aerobic glycolysis, producing lactate and pyruvate. These metabolites are then utilized by neighboring oxidative cancer cells for further metabolic activities. While the transmission of ROS in this process has been documented, the detailed mechanisms by which cancer cells and CAFs initiate and adapt to these metabolic changes remain less explored. Nevertheless, targeting the Reverse Warburg Effect presents a theoretical possibility to disrupt cancer metabolism, potentially offering new avenues for therapeutic intervention aimed at curbing tumor growth by altering the metabolic interplay between cancer cells and their stromal environment.

### CAFs mediate immunosuppression

Chronic inflammation, immune cell infiltration, and the ability of cancer cells to evade immune surveillance are recognized as key hallmarks of cancer progression (168). Research has demonstrated the dual role of the immune system in both suppressing and promoting tumor development, a phenomenon termed “cancer immunoediting.” This process involves three distinct stages: elimination, equilibrium, and escape (169).

During the elimination phase, innate and adaptive immune responses work in tandem to identify and destroy dysplastic cells before they progress to clinically detectable tumors. However, certain cancer cells may acquire immune-evasive or poorly immunogenic traits, allowing them to survive immune attacks and transition into the equilibrium phase. In this stage, the growth of neoplastic cells is restricted, and their immunogenicity is shaped under the selective pressure exerted by adaptive immunity, predominantly involving T cells and associated cytokines. Persistent immune selection pressure can lead these cancer cells to develop immunosuppressive or immune-evasive phenotypes, ultimately facilitating their progression to the immune escape phase. At this stage, cancer cells evade immune control entirely, resulting in unchecked growth, the formation of clinically evident tumors, and potentially metastasis (170). Despite significant progress, the complex mechanisms underlying cancer immunoediting remain incompletely understood, posing a barrier to developing effective immunotherapy strategies.

CAFs, as major components of the TME, play a pivotal role in promoting immune evasion. One of their key immunosuppressive mediators is TGF-β, which modulates the immune microenvironment by affecting T cell differentiation and proliferation through the inhibition of transcription factor activation triggered by Ca2+ influx (171). In ovarian tumors with T cell exclusion, elevated TGF-β expression and stromal activation are critical drivers of T cell exclusion. TGF-β reduces MHC-I expression on ovarian cancer cells *in vitro* and activates fibroblasts to promote extracellular matrix production, forming a physical barrier that impedes T cell infiltration (172). Additionally, TGF-β suppresses dendritic cell function, inhibits the development of cytotoxic natural killer (NK) cells and their secretion of IFN-γ, and polarizes macrophages towards an M2 phenotype with immunosuppressive, anti-inflammatory, and pro-angiogenic properties (173, 174). Beyond TGF-β, CAF-derived CXCL12 is a potent chemokine that modulates immune suppression. It restricts the migration of CD8+ T cells, sequestering them away from the tumor stroma, and inhibits NK cell proliferation, keeping them in a quiescent state (175, 176). Another significant CAF-secreted molecule in the immune microenvironment is IL-6, which is highly expressed in the inflammatory CAF (iCAF) subtype (176). IL-6 contributes to the accumulation of tumor-infiltrating lymphocytes and regulates neutrophil survival, activation, and function via the IL-6/STAT3/PD-L1 signaling axis (177, 178). Furthermore, CAFs secrete inhibitory immune checkpoints (iICPs) such as PD-1 and LAG3, further enhancing immune suppression within the TME (179).

These findings underscore the critical role of CAFs in facilitating tumor immune escape by creating an immunosuppressive microenvironment, thereby supporting cancer progression and resistance to immune-mediated therapies.

Non-follicular adaptive immune stimulating CAFs and Antigen-presenting CAF

In the context of cancer, non-follicular adaptive immune-stimulating CAFs and antigen-presenting CAFs (apCAFs) play distinct roles in modulating the immune response (180, 181). Non-follicular adaptive immune-stimulating CAFs, which predominantly reside outside organized lymphoid structures like cancer-associated tertiary lymphoid organs (CaTLOs), demonstrate a capacity to interact with the adaptive immune system beyond traditional immuno-suppressive roles (180). These CAFs are primarily characterized by their ability to activate and recruit adaptive immune cells rather than just innate immune interactions, marking a significant shift from the traditional view of CAFs as solely tumor-promoting entities (182).

Antigen-presenting CAFs (apCAFs), on the other hand, have a specialized role in direct immune modulation by presenting antigens to T cells via MHC class II molecules (116). CAFs expressing MHC class II, initially identified by Tuveson and colleagues in pancreatic cancer as apCAFs, have been subsequently confirmed by other research groups to be present in pancreatic, breast, and lung cancers (116, 183–187). This subset of CAFs is effective in initiating T cell responses, including the activation of effector CD4+ T cells (183–186). By presenting cancer antigens, apCAFs can influence T cell phenotypes and contribute to the immunological architecture of the tumor microenvironment (114). This function is critical for orchestrating localized immune responses against tumors and for supporting ongoing immune surveillance and anti-tumor activity.

Together, these roles underscore a complex interplay where CAFs can both suppress and stimulate immune responses, highlighting their dual potential as targets for cancer therapy (114, 188, 189). This emerging understanding challenges the traditional paradigm of CAFs and opens new avenues for therapeutic strategies aimed at modulating the tumor microenvironment to enhance anti-cancer immunity.

### CAFs facilitate cancer metastasis

Cancer metastasis is a multifaceted process encompassing various stages. It initiates with the migration and invasion of tumor cells into adjacent tissues, proceeds through intravasation into the bloodstream, follows by circulation and extravasation, and culminates in the colonization of new sites (190). CAFs significantly facilitate this process via paracrine signaling and direct physical interactions. The mobility of tumor cells, crucial for their migration and invasion, is often enhanced by the epithelial-mesenchymal transition (EMT), characterized by the loss of cell polarity and adhesion, which imparts a mesenchymal phenotype conducive to migration and invasion (191, 192). The necessity of EMT in all metastatic events remains debated.

CAFs are known to boost the migratory and invasive capabilities of cancer cells by secreting chemokines and exosomes. For instance, in gastric cancer, CAFs stimulated by TGF-β1/Smad2/3 signaling significantly upregulate hyaluronan and proteoglycan link protein 1 (HAPLN1), enhancing tumor migration and invasion (193, 194). In esophageal squamous cell carcinoma, CAF-like cells produce plasminogen activator inhibitor-1 (PAI-1), which augments migration and invasion through the PAI-1/low-density lipoprotein receptor-related protein 1 (LRP1) axis via Akt-Erk1/2 pathways. Furthermore, exosomes secreted by CAFs containing miR-18b and miR-382-5p have been documented to promote cancer cell migration and invasion through EMT induction (194). Additionally, CAFs facilitate EMT by modulating matrix stiffness and signaling pathways, such as the TWIST1/G3BP2 and EPHA2/LYN/TWIST1 pathways (195).

Apart from inducing EMT, CAFs also directly drive cancer cell migration through exerted physical forces. *Labernadie* and colleagues discovered a mechanism wherein CAFs apply physical force to cancer cells via heterophilic adhesion involving N-cadherin on CAFs and E-cadherin on cancer cells, mediated by β-catenin and α-catenin/vinculin interactions (196). *Erdogan* and team demonstrated that CAFs create and align a fibronectin-rich matrix, facilitating CAF-cancer cell association and directional migration through the nonmuscle myosin II/PDGFRα/α5β1-integrin/fibronectin pathway (197). Moreover, CAFs express membrane-anchored metalloproteinases (MT1-MMPs) that degrade collagen, easing tumor cell penetration and movement within the ECM (198).

Intravasation, a critical phase preceding circulation, involves tumor cells penetrating leaky, immature blood vessels formed during angiogenesis, often characterized by inadequate endothelial cell junctions and abnormal pericyte coverage (199). Various factors, including TGF-β, VEGF, and SOX2, have been identified as regulators of both intravasation and extravasation processes in metastasis (200, 201). CAFs enhance both hematogenous and lymphatic metastasis, with mechanisms involving signaling pathways such as periostin/integrin/FAK/Src/VE-cadherin, VEGFC/VEGFR3, and IL-6/IL-6R (202–204).

CAFs are dynamic participants in the metastatic process, not merely passive entities. For instance, circulating CAFs (cCAFs) found in the blood of patients with metastatic breast cancer correlate with clinical metastasis (205). These cCAFs, along with other cell types, form heterotypic clusters that influence survival and proliferation of circulating tumor cells (CTCs), potentially via soluble factors (206).

The target site microenvironment, often hostile to CTCs, is pre-emptively modified by the primary tumor, creating pre-metastatic niches (PMNs) (207). PMNs are shaped by cytokines and exosomes from the tumor and TME, with CAFs playing a dual role in their activation. CAF-derived factors, such as the non-coding RNA LncSNHG5 and extracellular vesicles, modify distant fibroblasts to enhance their tumor-supportive capabilities (208). Additionally, CAFs themselves undergo activation during PMN formation, further emphasizing their central role in cancer metastasis and providing potential therapeutic targets to hinder tumor growth and spread (209).

## CAF-targeting therapy

Recent years have witnessed significant advancements in therapies targeting cancer-associated fibroblasts (CAFs), focusing on three main objectives (1): directly or indirectly depleting CAFs, (2) mitigating or abolishing their tumor-promoting and immunosuppressive activities, or (3) reprogramming or normalizing CAFs towards a more dormant state. These strategies are outlined below.

### Chemotherapy targeting CAFs

Initially identified by Tuveson and colleagues, CAFs in various tumors express fibroblast activation protein (FAP), a unique membrane-bound serine postprolyl peptidase known for its additional endopeptidase activity (210). Val-boroPro (Talabostat), a competitive inhibitor of prolyl peptidase and an orally administered drug, demonstrated some control over tumor growth by degrading the extracellular matrix (ECM) in mouse models (211). However, it failed to show therapeutic efficacy in human clinical trials for metastatic colorectal cancers (212). Sibrotuzumab, a humanized anti-FAP monoclonal antibody, inhibited the dipeptidyl peptidase activity of FAP but did not show efficacy in suppressing pancreatic cancer growth in patients, despite the radiolabeled version of the antibody accumulating in tumors as visualized by SPECT (213, 214).

Utilizing FAP’s enzymatic activity, anti-CAF prodrugs or protoxins that couple cytotoxic agents with a dipeptide containing a FAP cleavage site have been developed (210, 215). These prodrugs, which remain inactive until cleaved by FAP upon systemic delivery, have induced tumor lysis and growth inhibition when injected intratumorally in human breast and prostate cancer xenografts (215). Similarly, immunotoxins like Anti-FAP-PE39 have suppressed tumor growth and enhanced recruitment of tumor-infiltrating lymphocytes (216). Other novel approaches include monoclonal antibodies conjugated with cytotoxic agents or bispecific antibodies that target both FAP on CAFs and death receptor 5 on tumor cells, showing potent antitumor effects (216).

### Immunotherapy targeting CAFs

Numerous approaches have been developed to bolster immunity against FAP-expressing cells, notably CAFs, and to curb cancer proliferation (2, 21, 180, 189, 217). Immunization using dendritic cells transfected with FAP mRNA has effectively curtailed the growth of both implanted and intravenously introduced tumors (218). This effect was amplified when the vaccine targeted both FAP and a tumor-associated antigen simultaneously. These dendritic cell vaccines, when used in conjunction with an anti-fibrotic agent, have effectively activated both innate and adaptive immune responses, leading to enhanced NK cell function, boosted anti-tumor humoral responses, and potentiated cytotoxic CD8+ T cell activity across diverse tumor models (218). Additionally, adenoviral vaccines targeting FAP have selectively eliminated CAFs by activating a CD8+ T cell response, thereby reducing tumor growth and metastasis in various murine cancer models (114, 189, 219). A significant study employing a transgenic mouse model engineered to express the diphtheria toxin receptor under the FAP promoter demonstrated that depletion of FAP+ CAFs via diphtheria toxin enhanced the efficacy of anti-cancer vaccines (220). Oral administration of an anti-FAP DNA vaccine markedly reduced new blood vessel formation, tumor growth, and metastasis in orthotopically injected breast carcinoma models (221), and the co-administration of doxorubicin significantly improved the drug’s intratumoral absorption and extended the survival of the treated mice (221).

Adoptive transfer of chimeric antigen receptor (CAR)-T cells specifically designed to target FAP-expressing cells has shown promise in depleting FAP+ populations, including CAFs, thus limiting tumor stroma formation and enhancing the effectiveness of chemotherapeutic agents (222–224). However, this strategy has been linked with severe adverse effects such as profound bone marrow toxicity and cachexia, underscoring the need for more selective targeting in CAF-based therapies, an area that continues to be vigorously researched (225). Furthermore, near-infrared photoimmunotherapy (NIR-PIT) represents a novel technique for selectively depleting FAP-positive cells within the tumor microenvironment, showing efficacy in inhibiting tumor growth in a co-culture xenograft model of human esophageal squamous cell carcinoma without negative side effects (226, 227). Combining anti-FAP+ CAF therapy with 5-fluorouracil (5-FU) has proven to surpass the effectiveness of 5-FU alone in overcoming chemoresistance (228).

### Functional modification/reprogramming targeting CAFs

Reverting activated CAFs to a quiescent state involves the use of agents like all-trans-retinoic acid (ATRA), minnelide (which disrupts the TGF-β signaling pathway), and calcipotriol (228–232). The angiotensin receptor II antagonist losartan has reduced TGF-β-mediated CAF activation, enhancing drug delivery and the efficacy of immunotherapy, and is being studied in clinical trials for pancreatic cancer treatment (98, 233–235). Efforts to block immunosuppressive ligands of key CAF signaling pathways, including IL-6 (185, 186), LIF (187), and TGF-β (124, 126) aim to suppress or eliminate cancer cells (236–240).

**Figure 4:**
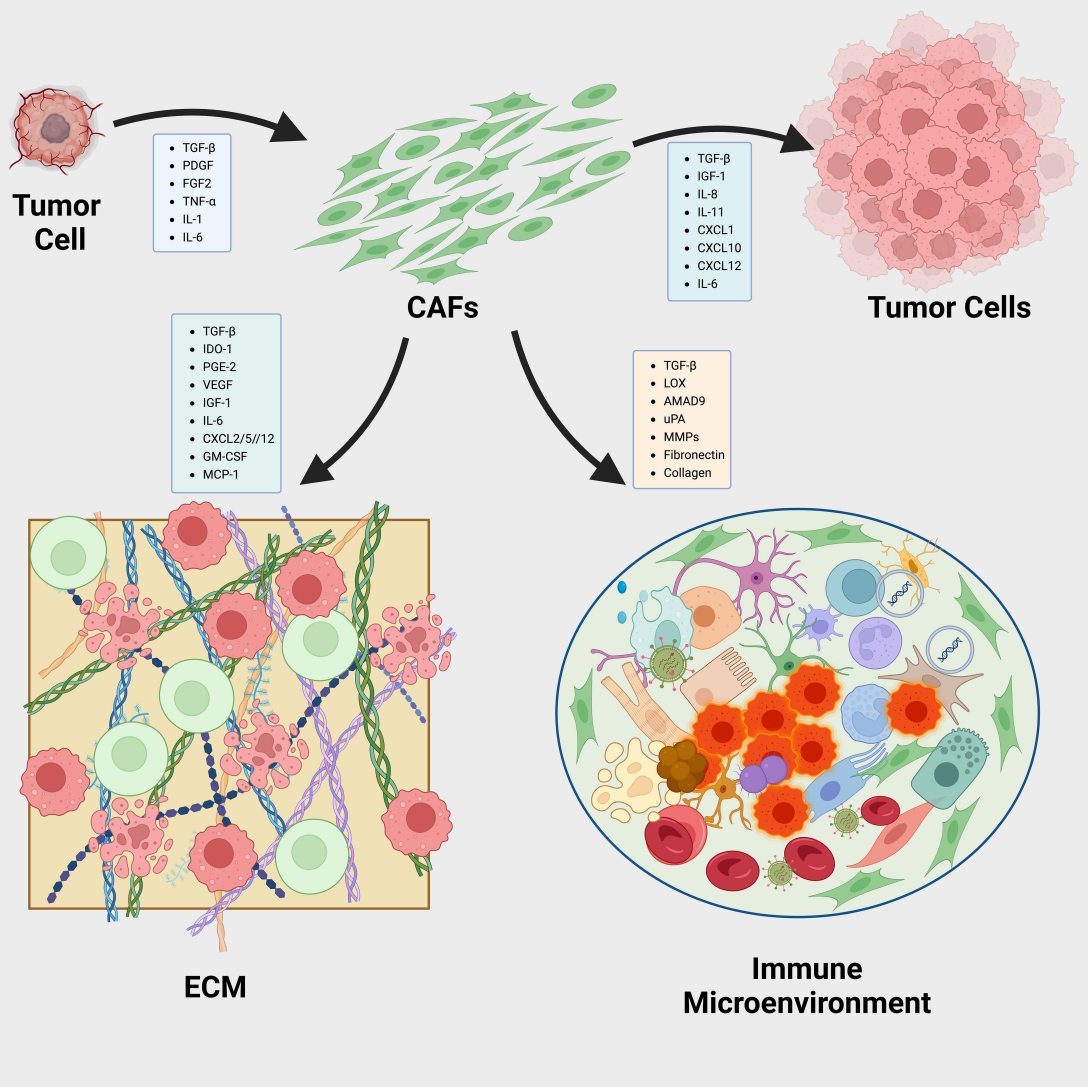
Dynamic Interactions Between CAFs and the Tumor Microenvironment in Cancer Progression. Reciprocal interactions between cancer-associated fibroblasts (CAFs) and the tumor microenvironment (TME) play pivotal roles in cancer progression via three primary mechanisms: a).Tumor-derived cytokines influence the behavior of CAFs, prompting them to release pro-tumorigenic factors that further drive the cancerous process. b).CAFs are instrumental in organizing and cross-linking the extracellular matrix (ECM) components, thus modifying the ECM’s structure and functionality to facilitate tumor growth and metastasis. c).Factors secreted by CAFs also modulate immune cells within the TME, including tumor-associated macrophages (TAMs), tumor-associated neutrophils (TANs), and dendritic cells (DCs). This modulation helps establish a tumor immune microenvironment (TIME) that promotes tumor development.

The CXCL12/CXCR4 axis, crucial in cancer progression and immunosuppression, involves CXCL12 from CAFs recruiting CXCR4-expressing cells that support angiogenesis and tumor growth (241–243). Inhibiting this pathway using the CXCR4 antagonist plerixafor has significantly reduced fibrosis and improved immune cell infiltration and checkpoint inhibitor efficacy (244). Other strategies that inhibit CAF functions include TGF-β blockade, NFkB inhibitors to overcome chemotherapy resistance, and Smoothened hedgehog pathway inhibitors (IPI-926) (102, 245–247) (**Figure 4**).

### Targeting CAF control cancer cell dormancy

Initial research has established cancer cell dormancy as a critical factor contributing to drug resistance and recurrence, yet dormant cancer cells (DCC) continue to be challenging to detect clinically, highlighting a significant obstacle in overcoming drug resistance (248). The process of dormancy entry and escape involves complex interactions between tumor cells and the TME, with cytokines and chemokines secreted by CAFs playing a vital role (249). Consequently, a promising strategy to prevent cancer recurrence involves targeting these CAF-secreted factors. This approach focuses on drugs that specifically target these factors, potentially inhibiting dormancy-associated mechanisms.

#### Inhibiting microenvironment interactions to prevent cancer cell reawakening

Maintaining tumor cells in a dormant state is critical to preventing metastasis and recurrence. It is imperative to develop therapeutic strategies that inhibit the communication between CAF-driven signaling and the supportive TME involved in dormancy escape (250). An effective target could be uPAR, which maintains the dormant state of cancer cells and limits metastasis (251, 252). For instance, ATN-292 reduces migration in human pancreatic cancer cells by blocking the uPA to uPAR binding (253), and a novel anti-uPAR monoclonal antibody has shown antitumor effects in gastric cancer by disrupting this interaction (254). The small molecule uPA inhibitor, WX-671, combined with gemcitabine, although well tolerated, did not improve survival outcomes compared to gemcitabine alone in a phase II trial (255).

High levels of TGF-β1 in the TME trigger dormancy escape, with CAFs being a primary source of TGF-β. Inhibitors targeting TGF-β1 interactions or receptor kinase activities are strategies to keep tumor cells dormant. Agents like SRK-181, which binds to the pro-segment of TGF-β1 preventing its activation, and LY2157299, a small molecule TGF-βRI kinase inhibitor known as Galunisertib, have shown promise in clinical trials (256–259). Another TGF-βRI inhibitor, Ki26894, has demonstrated efficacy in reducing invasiveness and bone metastasis in gastric cancer (260). Moreover, anti-inflammatory therapies targeting pro-inflammatory cytokines secreted by CAFs have been studied. NSAIDs such as sulindac and celecoxib, which inhibit COX-2 activity, have shown efficacy in gastrointestinal cancers in both preclinical and clinical settings. Sulindac is under investigation in a phase III trial for its potential to reduce adenomas and secondary cancers (261, 262), and celecoxib is being studied to enhance response rates in advanced colorectal cancer treatment (263, 264).

CAF-mediated ECM remodeling significantly contributes to dormancy escape. Targeting ECM molecules like collagen and FN, which regulate integrin roles in dormancy to proliferation transitions, is a potential strategy. Anti-integrin therapies like Volociximab have shown positive results in clinical trials (265, 266).

Lastly, inhibiting enzymes like LOX or LOXL2, which are implicated in chemoresistance and metastasis growth, could prevent dormant cancer cell awakening. Agents like Simtuzumab and EGCG have been evaluated for their efficacy in reducing LOXL2 activity and TGF-β1 signaling, showing potential in clinical trials (267, 268).

In conclusion, reinforcing the dormant state and inhibiting the pathways facilitating dormancy escape through targeted therapies offers a promising avenue for managing cancer progression and recurrence.

#### Activate dormant cancer cells for improved treatment response

Understanding cancer dormancy has led to strategies aimed at preventing cells from becoming dormant or awakening dormant cancer cells (DCCs) to increase their sensitivity to treatment. One approach involves targeting dormancy-inducing factors influenced by CAFs. For instance, TGF-β2, which promotes dormancy via the TGF-βRIII pathway, is targeted by AP 12009 (Trabedersen), an antisense oligonucleotide (ASO) that has shown safety in phase I/II studies for pancreatic and colorectal cancer (269, 270).

Another CAF-secreted factor, DKK-1, helps maintain cancer cell dormancy (271). DKN-01, a humanized monoclonal antibody that inhibits DKK-1, is currently being evaluated in clinical trials for gastrointestinal (GI) cancers (272, 273). Notably, a phase II trial is investigating DKN-01 in combination with Tislelizumab and possibly chemotherapy for metastatic gastric cancer or gastroesophageal junction adenocarcinoma (274). Additionally, DKN-01 is being tested with pembrolizumab in advanced esophageal cancer in a phase Ib trial and in combination with bevacizumab and chemotherapy for advanced colorectal cancer in another ongoing phase II study (NCT05480306) (272, 275).

**Figure 5:**
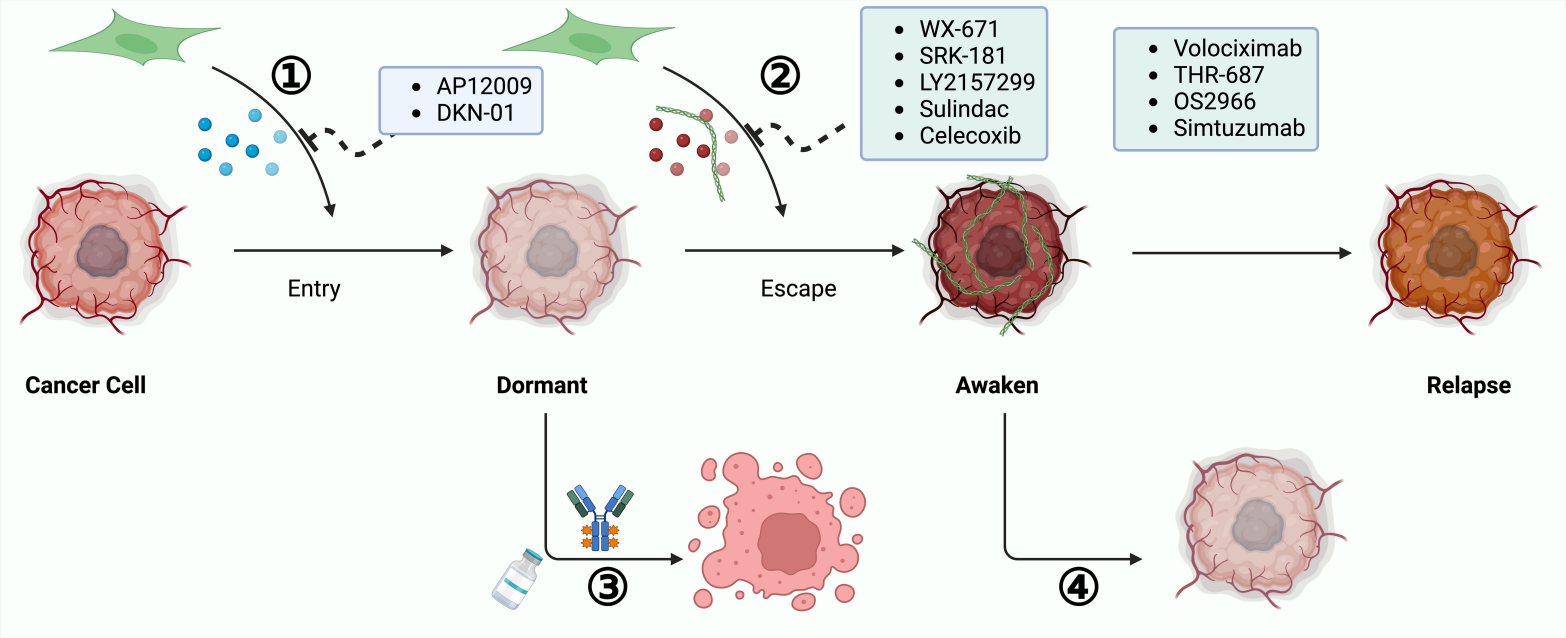
Approaches to Combat Cancer Recurrence by Targeting Dormant Cancer Cells. Targeting dormant cancer cells presents a viable strategy for preventing cancer recurrence. 1). Reactivating Dormant Cancer Cells for Therapeutic Sensitivity: This involves awakening dormant cancer cells to increase their susceptibility to treatments. Strategies include obstructing the secretion of dormancy-inducing factors by CAFs or reactivating cellular proliferation signals. 2).Sustaining Permanent Dormancy of Cancer Cells: This strategy aims to prevent cancer cell reactivation and subsequent growth by blocking pathways facilitated by CAFs that enable dormancy escape. By focusing on these dormant cancer cells, it is possible to avert cancer recurrence and enhance patient outcomes.

Other factors like GDF-10 and BMP4, also secreted by CAFs, have been implicated in promoting dormancy, although targeted therapies for these factors in GI cancers have yet to be explored (276–278). By inhibiting these CAF-derived factors, it is possible to either prevent entry into or trigger exit from dormancy. Implementing such strategies early in treatment may prevent tumor cells from developing robust malignancy. A summary of targeted factors by therapeutic agents in GI cancers emphasizes potential approaches to manage cancer dormancy at various stages (**Figure 5**).

## CAFs and therapeutic resistance

**Figure 6:**
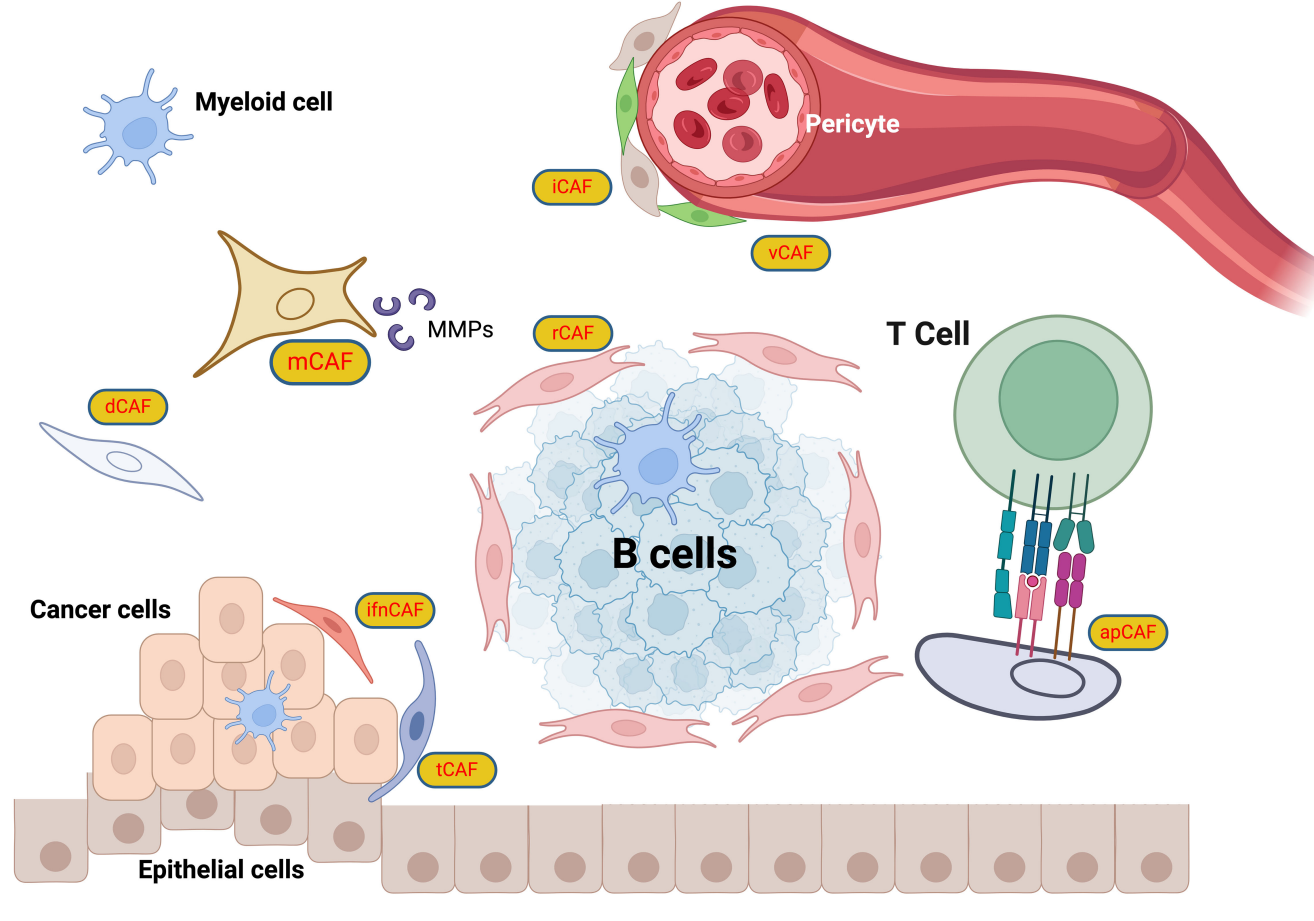
CAF classification scheme. The CAFs are categorized into specific roles: iCAFs (inflammatory CAFs) that modulate inflammation within the TME (286); rCAFs (regulatory CAFs), which might play roles in tumor regulation (2); dCAFs (developmental CAFs) associated with developmental processes (103); tCAFs (TGF-β producing CAFs), known for TGF-beta production influencing tumor growth and immune evasion (287); vCAFs (vascular CAFs) involved in vascular dynamics (288); ifnCAFs (interferon-producing CAFs), which might interact with immune pathways through interferon production (289); and apCAFs (antigen-presenting CAFs) that potentially present antigens to T cells, facilitating immune system interactions (290). This classification highlights the multifunctional nature of CAFs, underlining their importance in tumor progression, immune modulation, and the structural integrity of tumors, thus providing crucial insights for targeting these cells in cancer therapy strategies.

Resistance to cancer therapy often results in poor patient outcomes, underpinned by complex and dynamic mechanisms. Konieczkowski et al. introduced a convergence-based framework to understand cancer drug resistance, identifying major causes such as pathway reactivation, pathway bypass, and pathway indifference (279). Beyond genomic alterations in tumor cells, the role of CAFs in therapeutic resistance has been well-established, with their influence extending across multiple facets. CAFs affect the mechanical properties of the tumor microenvironment (TME), enhancing matrix stiffness which can impede the penetration of chemotherapeutic drugs. For example, gastric CAFs that express calponin 1 activate the ROCK1/MLC pathway, increasing matrix stiffness and contributing to resistance against 5-fluorouracil (5-FU) in cancer cells by activating the YAP signaling pathway (280). CAF-derived exosomes are also pivotal in mediating resistance within the TME (281). Annexin A6 in CAF-derived extracellular vesicles (EVs) activates the integrin β1-FAK-YAP signaling pathway, promoting the formation of a tubular network in the ECM that reinforces chemotherapeutic resistance (282). In breast cancer, CAF-derived circulating EVs containing the full mitochondrial genome enhance estrogen receptor (ER)-independent oxidative phosphorylation (OXPHOS), which induces therapy-resistant dormant cancer stem-like cells, leading to resistance to endocrine therapy (283). Targeting the YAP signaling pathway may be effective in overcoming the mechanical resistance encountered in targeted therapy. Regarding immunotherapy, CAFs activated by the IL-17/Act1/HIF1α pathway can lead to collagen deposition, enhancing PD-L1 resistance and reducing cytotoxic T cell infiltration (284). Another subtype of CAF, ecm-myCAF, has been shown to elevate PD-1 and CTLA4 protein levels in Tregs, boosting TGFβ-myCAF cellular content and mediating primary resistance to immunotherapy. Combining tumor-targeted therapy with CAF-targeted strategies, such as the FAP5-DM1 monoclonal antibody conjugated to maytansinoid, has demonstrated prolonged inhibition of tumor growth and complete regressions in xenograft models of multiple cancers (222). Additionally, CAFs contribute to radiotherapy resistance; upon irradiation, CAFs polarize towards the iCAF subtype via IL-1a, leading to oxidative DNA damage and p53-mediated therapy-induced senescence in iCAFs, which in turn facilitates chemoradiotherapy resistance and disease progression (285) **(** **Figure 6** **)**.

## Challenges and directions

The challenges surrounding CAFs are multifaceted, ranging from classification issues to their dynamic roles within the TME. One major challenge lies in the lack of a uniform and comprehensive naming standard for CAF subgroups. An ideal naming convention should consider factors such as cell lineage, functional roles, biomarkers, clinical correlations, immune regulation, immune response, and metabolic status, integrating all these aspects to advance our understanding of CAFs. Another obstacle is the difficulty in identifying the origin of CAFs, which is compounded by the absence of specific biomarkers. A promising approach to this issue would involve the combined use of multiple biomarkers and the quantitative assessment of their variations to enhance specificity, particularly by focusing on distinct CAF subpopulations. Moreover, *in vitro* culture of CAFs presents significant challenges, as most of their *in vivo* characteristics tend to change due to alterations in culture conditions and passage (291, 292). I t is crucial to establish culture environments that closely mimic the TME and to track phenotypic changes during cultivation to preserve CAF traits. Despite the use of various methods for detecting CAFs, including antibodies, mRNA probes, and single-cell transcriptome analysis, there is still a lack of standardized, accurate, and universally applicable quantitative methods for their detection. While single-cell transcriptomics is already shedding light on CAF heterogeneity, further application of this technology is essential for deepening our understanding of CAF subpopulations (293). Another critical gap is the lack of longitudinal studies examining CAFs across different experimental stages, such as primary tumor growth, early isolation, and long-term passage, as well as across varying clinical stages (294). Such studies are necessary to improve our knowledge of CAF origins, subpopulations, heterogeneity, and plasticity in relation to tumor progression. Additionally, there is a need for horizontal studies that compare CAF subpopulations between different types of tumors and correlate these populations with clinical features to better understand the impact of CAFs on disease progression and treatment responses (2).

The regulation of CAFs within the TME is also not fully understood, and a deeper exploration of the dynamic interplay between CAFs, tumor cells, and other elements of the TME from biochemical, metabolic, immunological, and physical perspectives is necessary. Understanding how CAFs evolve in response to tumor progression and TME changes is equally crucial. Furthermore, while CAFs are known to influence immune responses within the TME, their crosstalk with immune cells remains poorly defined, highlighting the need for more research into how CAFs contribute to immune evasion and therapy resistance.

## Discussion

CAFs represent a crucial, yet complex, component of the TME, influencing various aspects of cancer progression, metastasis, and therapeutic resistance. This review has highlighted the multifaceted roles of CAFs in shaping the TME, including their involvement in ECM remodeling, immune modulation, angiogenesis, and metabolic reprogramming. The dynamic and heterogeneous nature of CAFs, however, complicates their classification and therapeutic targeting. Current research on CAFs has emphasized the need for a more standardized system to categorize the various CAF subpopulations based on their lineage, function, biomarkers, and interactions within the TME. Understanding the underlying mechanisms of CAF activation and their dualistic roles—either promoting or suppressing tumor growth depending on the context—remains a critical challenge for therapeutic strategies.

To effectively address the highlighted lack of specific CAF markers, concerted efforts are needed to identify and validate reliable markers that enhance specificity. This can be achieved by employing advanced genomic and proteomic technologies to analyze diverse cancer types, facilitating the discovery of unique CAF profiles. Additionally, the lack of specific biomarkers for CAF identification remains a significant hurdle, limiting their effective targeting in clinical practice. Although several biomarkers have been proposed, the lack of specificity for CAFs means that they cannot be universally applied in clinical settings. The use of advanced techniques, such as single-cell transcriptomics, holds promise for resolving the complexity of CAF subpopulations and identifying precise markers for their targeting. Another critical issue need to be discussed is the challenge of maintaining CAF phenotypes *in vitro*, as their characteristics often change when cultured outside the TME. Optimizing culture conditions to better preserve the *in vivo*-like properties of CAFs is crucial for advancing CAF-based research and therapeutic development.

Single-cell technologies have profoundly refined our understanding of CAFs by revealing their cellular heterogeneity within the tumor microenvironment. Techniques like single-cell RNA sequencing (scRNA-seq) have identified distinct CAF subtypes with unique gene expressions and roles, enhancing our insight into their contributions to tumor progression and potential as therapeutic targets. Future research should integrate single-cell data with spatial transcriptomics to explore the dynamic interactions of CAFs with the TME across tumor development and therapy response, aiming to develop targeted treatments that disrupt crucial CAF-driven pathways.

The resistance mechanisms mediated by CAFs, particularly in the context of chemotherapy, immunotherapy, and radiotherapy, further complicate treatment efficacy. CAFs influence drug resistance through several mechanisms, including the promotion of ECM stiffness, secretion of pro-inflammatory cytokines, and modification of immune responses within the TME. Notably, CAFs enhance immune evasion by modulating the activity of immune cells, such as Tregs and NK cells, which significantly impacts the success of immunotherapies. Therefore, strategies that combine CAF-targeted therapies with conventional treatments, such as chemotherapy or immune checkpoint inhibitors, may offer a promising approach to overcome therapeutic resistance.

In addressing the current challenges and gaps identified in CAF research, we propose several specific experimental approaches and therapeutic strategies to advance this field. Firstly, the development of innovative CAF-specific markers through high-throughput screening techniques could greatly refine the targeting of these cells in diverse cancer types. Additionally, leveraging cutting-edge technologies such as CRISPR-Cas9 for gene editing within CAFs offers a promising avenue to dissect their functional roles in tumor progression and resistance mechanisms (295). Therapeutically, exploring bi-specific antibodies that target both CAFs and tumor cells could provide a dual approach to disrupt the supportive tumor microenvironment. Furthermore, employing organoid models incorporating CAFs from patient-derived samples would enhance our understanding of their interaction with tumor cells in a controlled, yet biologically relevant system. These approaches not only aim to fill the existing gaps but also pave the way for novel interventions that could be translated into clinical applications.

However, further research is still essential to gain a deeper understanding of the molecular mechanisms that regulate CAF behavior, particularly their interactions with tumor cells, immune cells, and the extracellular matrix. Investigating the dynamic roles of CAFs in different stages of tumor progression, as well as their involvement in the establishment of pre-metastatic niches, will be crucial for developing more effective therapeutic strategies. Additionally, refining methods to accurately identify and classify CAF subpopulations, along with developing therapies that can specifically modulate CAF function, holds great promise in advancing cancer treatment. Combining CAF-targeted therapies with current immunotherapies and other treatment modalities could significantly improve clinical outcomes and provide more effective treatment options for cancer patients.
